# Schistosomiasis in Ghana from baseline to now: the impact of fifteen years of interventions

**DOI:** 10.3389/fpubh.2025.1554069

**Published:** 2025-06-06

**Authors:** Joseph Opare, Tei Hervie, Ernest Mensah, Charles Brown-Davies, Odame Asiedu, Bright Alomatu, Ebenezer Padi Ako, John Frederick Dadzie, Irene Dzathor, Vance Harris, Julie Ritter, Darin Evans, Anna Elizabeth Phillips

**Affiliations:** ^1^Neglected Tropical Diseases Programme, Ghana Health Service, Accra, Ghana; ^2^Family Health International (FHI 360), Accra, Ghana; ^3^Family Health International (FHI 360), Washington, DC, United States; ^4^United States Agency for International Development (USAID), Washington, DC, United States

**Keywords:** *Schistosomia haematobium*, *Schistosomia*, *Mansoni schistosomiasis*, neglected tropical disease, Ghana, school - aged children

## Abstract

**Background:**

Schistosomiasis is a major public health problem in Ghana, significantly impacted by the construction of dams during the 1960s that resulted in the creation of Lake Volta. The Ghana Health Service launched the Neglected Tropical Disease program in 2008, expanding baseline disease mapping first initiated in 2007 to additional geographic areas in 2008 and 2010 and simultaneously rolling out mass drug administration (MDA). Significant reduction of infection across the country was noted in 2015 during the first impact assessment, following five years of MDA. After another five years of treatment, a second nationwide survey has been rolled out since 2021 to re-evaluate the situation of schistosomiasis this time at the sub-district level.

**Methods:**

Prevalence at three time points is presented. At baseline (2007–2010), a cross-section of 13,299 school-aged children (SAC) were tested from 251 schools across 154 districts in Ghana. During the first impact assessment (2015), 156 schools were sampled across 114 districts, with a total of 7,803 SAC tested. More recently, a second impact evaluation (2021-2024) has been rolled out across 1,146 schools sampled in 61 districts, with a total, 29,924 SAC tested. In all surveys, urine samples were filtered for the presence of *Schistosoma haematobium* eggs, with haemastix® testing conducted in recent surveys only, and Kato-Katz performed on each stool sample for the presence of *Schistosoma mansoni*.

**Results:**

At baseline, overall schistosomiasis prevalence was 21.1% (95% CI 17.0–26.0), with 20.4% (95% CI 16.4–25.2) *S.haematobium* and 1.01% (95% CI 0.55–1.84) *S.mansoni.* Prevalence of schistosomiasis decreased dramatically at first impact assessment, with overall prevalence at 3.5% (95% CI 2.6–4.7) and remained low in recent surveys at 6.8% (95% CI 6.1–7.6), which represents a 67.8% reduction from baseline to current prevalence.

**Conclusion:**

After over a decade of treatment since 2008, Ghana has made significant progress in reducing the burden of schistosomiasis infection. Indeed, the most recent surveys demonstrated that elimination as a public health problem (heavy intensity of infection <1%) has been achieved in 75.4% districts surveyed, which is a considerable achievement. Furthermore, recent assessments have been conducted at the sub-district level, which has therefore enabled the Ghana Health Service to change to a more focal intervention and therefore tackle morbidity in the remaining high transmission zones.

## Introduction

Schistosomiasis, commonly known as bilharzia, is a neglected tropical disease (NTD) affecting an estimated 251.4 million people across 51 countries, with over 90% of cases in Africa (1–3). Schistosomiasis is an acute and chronic parasitic disease caused by blood flukes (trematode worms) of the genus *Schistosoma*. Five species of Schistosoma are responsible for human schistosomiasis, globally, the three most common are *Schistosoma mansoni* and *S. japonicum* and *S. haematobium* with the former two causing intestinal and latter urogenital schistosomiasis. Schistosomiasis is particularly high in settings with poor sanitation and limited safe-water infrastructure and access (4, 5).

The global target set in World Health Organization (WHO) roadmap on NTDs is to reach at least 75% of all school-age children (SAC) who are at risk of morbidity from schistosomiasis (3, 6). The main control strategy used by the majority of the African Region is to first control the morbidity then eliminate schistosomiasis as a public health problem through mass drug administration (MDA) with praziquantel (7, 8). In 2022, WHO released new treatment recommendations for annual MDA shifting from a school-based focus to among all age groups above two years of age in communities with prevalence of schistosomiasis ≥10%, including pregnant women after the first trimester and lactating women. In communities with schistosomiasis prevalence <10%, WHO suggests one of two approaches based on programmatic objectives and resources: (i) where there has been a programme of regular MDA, continue treatment at the same or reduced frequency; or (ii) where there has not been a programme of regular preventive chemotherapy, to use a clinical approach of test and-treat, instead of MDA (6). There is, however, a global limited availability of donated praziquantel that is not sufficient to treat all age groups in every moderately/high endemic district. In attempt to free up more drugs for those in need, there is simultaneous shift from mass treatment at the district to sub-district, but prevalence data often does not exist at this level and so countries, such as Ghana, have been conducting sub-district assessments to fill this evidence gap.

Schistosomiasis is of major importance as a public health problem in Ghana, likely due to the construction of the Akosombo hydroelectric dam during the 1960s that resulted in the creation of Lake Volta (approximately 8,500 km^2^), suitable for the breeding of freshwater snails that serve as intermediate hosts of schistosomiasis. Indeed, the prevalence of *S. haematobium* rose from 5–10% (before the dam) to >90% in many communities along Lake Volta (9–13). Similarly, construction of agricultural dams in the Upper East Region in the 1960’s resulted in a rise of *S. haematobium* from 17 to 51% (14). The Ghana Health Service (GHS) Neglected Tropical Disease program (NTDP) was launched in 2008, which coincided with the nationwide mapping of schistosomiasis and soil-transmitted helminths (STH) (15). Schistosomiasis prevalence mapping is essential to determine a specific treatment regimen and within an implementation unit, and national teams in Ghana started surveys to map schistosomiasis in 2007. Since it is not possible to assess every community, or even every district in Ghana given the high number, geospatial risk mapping was applied to the parasitological mapping data collected in 2008 to predict prevalence in unsampled locations to predict prevalence and intensity of infection throughout the country (16). The findings confirmed the earlier assessment that the geographical distribution of *S. haematobium* in Ghana was highly heterogeneous but endemic in all districts and more widespread than that of *S. mansoni*.

The NTDP then started treatment in 2008, with support from the U.S. Agency for International Development (USAID) (14). In 2010, additional schools that were not sampled during 2007–2008 were surveyed by the GHS, which enabled MDA to roll out in all 170 districts in 2011. Following five years of treatment, Ghana’s first schistosomiasis impact assessment was conducted in 2015 including many of the same districts surveyed at baseline. The impact survey demonstrated that the epidemiological profile of infection in these endemic areas in Ghana has changed considerably thanks to successful preventive chemotherapy conducted through school platforms. Following another five rounds of MDA, Ghana have been scaling up further surveys since 2021 to not only evaluate the impact of MDA and reassess the burden of schistosomiasis that is recommended after five years of MDA, but also estimate prevalence at the sub-district level to enable the shift in implementation strategy (6, 17). Here we present the prevalence and intensity of schistosomiasis among SAC in Ghana from baseline to a first impact assessment (2015), and finally more recently (2021–2024) following ten rounds of preventive chemotherapy implemented since the start of the schistosomiasis control program.

## Methods

### Study population

Ghana has a population of over 34 million people in 2023, all of whom are at risk for schistosomiasis (18). There have been several national scale surveys conducted over the last fifteen years of interventions that has demonstrated this endemicity. In brief, at the time of baseline mapping between 2007 and 2010, there were 170 districts in the country, which was restructured in 2012 and 2019 bringing the total to the current 261 districts, with a total of 1,405 sub-districts. To date, 202 districts (554 sub-districts) have been surveyed for schistosomiasis and all 261 districts have been treated with praziquantel MDA. Here we present the methods and results of these surveys, by year, and change in infection burden over time.

### Study design and sampling

There were three major surveys that have been conducted in Ghana over the last fifteen years, the first baseline evaluation was carried out between 2007 and 2010 followed by a post-intervention evaluation in 2015, and more recently a second re-assessment survey in 2021–2024. Over this period that has been a change in focus by WHO (from district to sub-district implementation) and donors (from Gates Foundation to UKAID to USAID, all of whom have different budget priorities). This has influenced consistency of study design, which has been different for each of these surveys (Table 1).

**Table 1:** Summary of schistosomiasis surveys from baseline mapping to the first and second impact assessment surveys.

Year of survey	Survey	Survey design	Number of schools sampled/district	Number of SAC sampled/ school	Diagnostic
2007–2010	Baseline mapping	Stratified, two-stage cluster survey at the district level	Proportional to size of the district	60	Single Kato-Katz and Urine Filtration
2015	First impact assessment	Stratified, two-stage cluster survey at the district level	Proportional to size of the district	50	Single Kato-Katz and Urine Filtration
2021–2024	Second impact assessment	SOS survey: compact segment sampling Stratified, two-stage cluster survey at the sub-district	40% communities across three contiguous districts 30–40% communities per sub-district	30 24	Duplicate Kato-Katz and Haematuria and Urine Filtration

#### Nationwide baseline mapping (2007–2010)

A cross-sectional design using a stratified, two-stage cluster survey strategy estimated prevalence among SAC at the district level whereby the number of schools selected was proportional to the size of the district (15, 19, 20). In brief, a total of 60 SAC, aged 5–14 years, were randomly sampled per school stratified equally by sex (30 girls and 30 boys).

#### First impact assessment (2015)

The first impact survey was conducted to investigate the impact of five rounds of MDA since the start of the program. Selection of survey sites was based on criteria from previous survey work conducted in 2008, with 50 SAC aged 5–14 years selected per school. In both baseline mapping and the first impact assessment, each child was asked to provide a stool and urine sample that was, respectively, examined microscopically using single Kato-Katz for *Schistosoma mansoni* and 10 mL of urine was filtered through a polycarbonate membrane for *S. haematobium*.

#### Second impact assessment (2021–2024)

As ten years of MDA had been conducted and simultaneously the WHO had changed their focus from district to sub-district implementation unit, the survey was designed to estimate the prevalence of schistosomiasis at the sub-district level. There are two strategies used during this time: first the multi-country Schistosomiasis Oversampling Study (SOS) conducted in 2021–2022 (21). In brief, the SOS method randomly sampled 40% of communities across three contiguous districts, with 30 SAC recruited per community using compact segment sampling. An additional 18 communities were systematically selected to address areas with low recruitment rates. The second survey conducted between 2022 and 2024 used stratified, two-stage cluster survey strategy to estimate the prevalence of schistosomiasis at the sub-district level. Survey sample size was decided based on precision-based sample size calculations, which since the sub-district is less ecologically heterogeneous than the district, used an estimated intra-cluster correlation coefficient (ICC) of 0.041 resulting in 15–20 schools per district or 30–40% of communities per sub-district, with 24 children sampled per school (12 girls and 12 boys) aged 5–14 years (22). Sample size calculations could estimate 50% prevalence with a 10-percentage point margin of error on the 95% confidence interval, using calculations from Lohr (2009) (23).

Makeshift laboratories were set up in the schools with samples tested immediately with urine dipsticks (Haemastix®) for the presence of blood, as a proxy measure for *Schistosoma haematobium*. The Haemastix® results were graded as negative, trace non-haemolysed, trace haemolysed, +, ++ and +++. All haematuria positive (trace haemolysed, +, ++ and +++ only) samples were then processed using the filtration technique for *S. haematobium* eggs per 10 mLs of urine. Infection was categorized according to the WHO guidance where heavy infections were defined as ≥50 eggs per 10 ml urine (7). Urogenital schistosomiasis infection was defined as having the presence of haematuria and/or *S. haematobium* eggs in the urine. Stool samples were examined for the presence of *Schistosoma mansoni* using duplicate Kato-Katz on a single stool sampled. Average eggs across two slides were used to calculate the eggs per gram (epg) of stool for each organism and the threshold for heavy-intensity infections was ≥400 epg of stool. Infection of any schistosomiasis was defined as presence of haematuria, *S. haematobium* eggs in the urine and/or *S. mansoni* in stool. About 10% of the slides were re-read by the supervisor to give the teams that assurance of quality.

### Data management and analysis

Prior to data collection, information was sent to the selected school authorities to seek consent from parents and assent for the children to participate in the study. Only consented parents and assented children were recruited into the study. Each child was asked to provide information on their age, sex, and a unique ID number was allocated to them. This unique ID number was written down by the data collector onto a urine and stool pot and given to the child to provide a sample for parasitological screenings. The head teachers were interviewed to get information on the water and sanitation status. In a selected school, students from the first six primary school classes were recruited into the study. To select the SAC, all students who had a signed parental consent were assembled in two lines, one for boys and one for girls. Children were selected systematically from within these two lines, using a sampling interval derived from the target sample size and the number of children. A list of the students who were selected for the study were given to the school authority for their records. Global positioning system (GPS) coordinates for each school were recorded.

In the baseline mapping and first impact assessment data was collected on paper and individual level data then double data entered into Microsoft Excel (Microsoft Corporation, Bellevue, WA). In the second impact assessment, data were collected on Android tablets using KoboCollect application v2021.2.4. School and student-level data were linked to their parasitological results using unique barcode identifiers. Analysis utilized current geographic administrative jurisdictions to ensure effective comparisons over time. District-level prevalence was estimated as the proportion of SAC who tested positive for schistosomiasis out of the total number tested. The sampling and survey design at each time period facilitates a direct calculation approach for district-level prevalence. Country and region-level prevalence estimates, 95% confidence intervals, and prevalence comparisons for any schistosomiasis, *S. mansoni*, and S. haematobium were calculated using negative binomial regression. These models utilized school-level aggregated data, with the outcome defined as the number of SAC testing positive for schistosomiasis, offset by the number of SAC tested. Negative binomial regression was chosen to account for overdispersion in the count data, which is common in schistosomiasis prevalence due to high variability between schools and geographic areas. Only summarized school-level data was available for baseline, however, individual-level data including gender, age, and intensity of infection was available and analyzed for the first (2015) and most recent (2021–2024) impact assessments, using the same methods described. Data was analyzed in SAS 9.4 (Cary, NC, USA) with figures generated utilizing multiple packages in R (v4.3.3; R Core Team 2024, Vienna, Austria). The maps are created utilizing Python (Python Software Foundation, version 3.13.1. Available at http://www.python.org) to join the prevalence tables with the shapefiles and ArcGIS Pro to visualize the data and create the maps.

## Results

### Baseline mapping (2007–2010)

In 2007, 51 schools across 48 districts were surveyed, with a total of 2,539 SAC providing samples. In 2008, 77 schools were sampled across 63 districts with a total of 4,610 SAC providing samples. In 2010, 123 schools were sampled in 92 districts with samples from 6,150 SAC. Over the entire baseline mapping period, this resulted in a total of 13,299 samples representing 251 schools across 154 currently defined districts in 16 regions (Table 2).

**Table 2:** Number of survey regions, districts, subdistricts, schools, and samples collected during schistosomiasis mapping and impact assessment surveys (utilizing current administrative jurisdictions).

Survey time period	Number of regions	Number of districts	Number of subdistricts	Number of schools	Number of samples
2007–2010 Baseline mapping	16	154	222	251	13,299
2015 Impact assessment	16	114	146	156	7,803
2021–2024 Impact assessment	10	61	337	1,146	29,924

**Figure 1:**
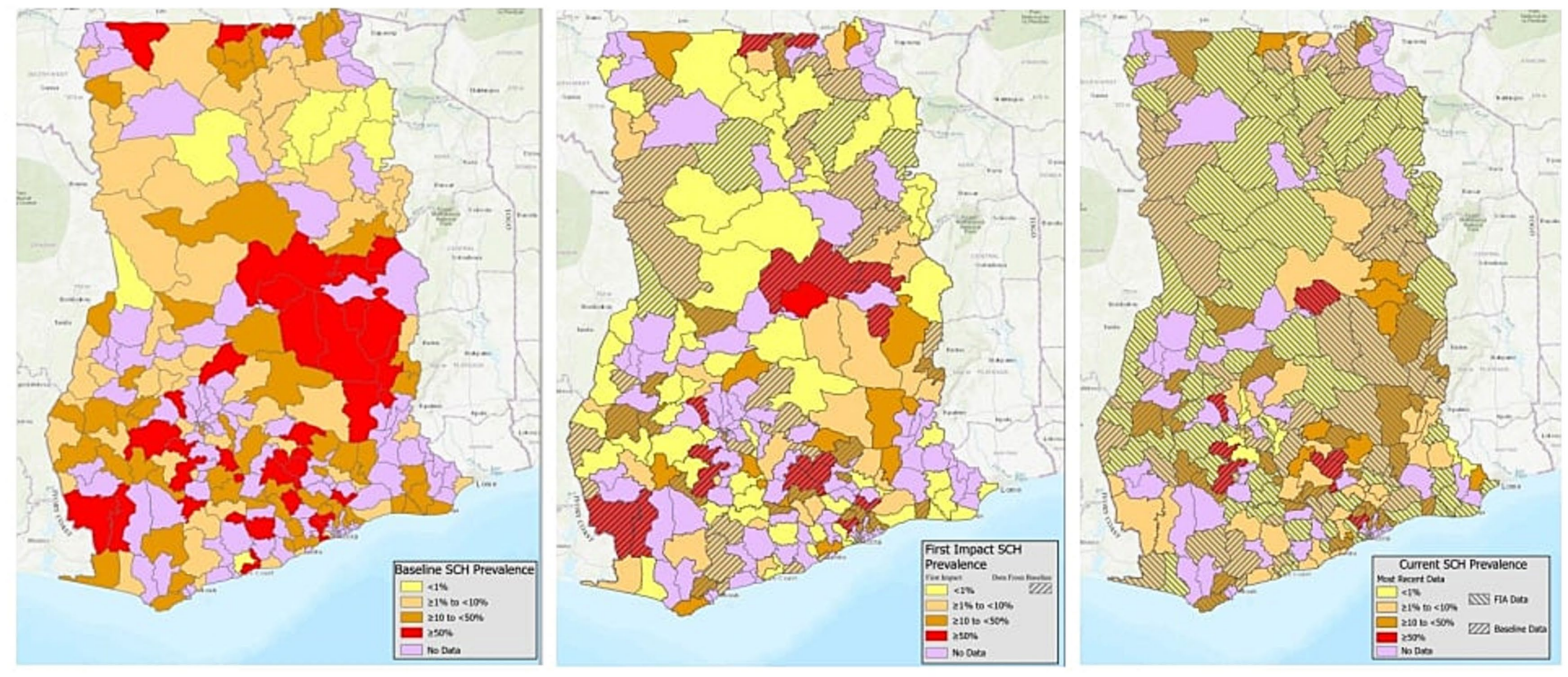
District schistosomiasis prevalence at baseline (2007–2010), first impact assessment (2015), and most recent impact assessment (2021–2024).

Overall schistosomiasis prevalence at baseline was 21.1% (95% CI 17.0–26.0), where the predominant species is *S.haematobium* with overall baseline prevalence of 20.4% (95% CI 16.4–25.2) and *S.mansoni* was only 1.01% (95% CI 0.55–1.84) (Table 3). All regions surveyed at baseline had at least some schistosomiasis, however, district level prevalence ranged from 0 to 98.3% (Figure 1; Supplementary Table 1).

**Table 3:** Comparison of schistosomiasis prevalence and intensity, total and by species, at baseline mapping and two impact assessments.

Survey time period	Any SCH % (95% CI)	*Schistosoma mansoni* % (95% CI)	*Schistosoma haematobium* % (95% CI)
Prevalence % (95% CI)			
2007–2010 Baseline mapping	21.05 (17.02–26.03)	1.01 (0.55–1.84)	20.35 (16.40–25.24)
2015 Impact assessment	3.48 (2.60–4.66)	0.27 (0.11–0.63)	3.28 (2.44–4.41)
2021–2024 Impact assessment	6.78 (6.09–7.56)	0.12 (0.08–0.20)	6.72 (6.02–7.50)
**Relative reduction (*p*-value)**	**67.79% (<0.0001)**	**88.12% (<0.0001)**	**66.98% (<0.0001)**
Heavy intensity prevalence % (95% CI)**			
2015 Impact assessment	1.61 (0.92–2.86)	0.27 (0.11–0.67)	1.40 (0.82–2.40)
2021–2024 Impact assessment	2.11 (1.70–2.62)	0.07 (0.04–0.12)	2.55 (2.05–3.18)
**Relative reduction**	**131.06% (0.39)**	**74.07% (0.02)**	**182% (0.04)**

*Relative reduction in prevalence from baseline mapping (2007–2010) to most recent impact assessment (2021–2024).

**Intensity data was not available in the baseline mapping dataset.

#### First schistosomiasis impact assessment (2015)

In total, 156 schools were sampled across 114 districts, with a total of 7,803 SAC providing a stool (7,747) and/or urine sample (7,781) (Table 2). Prevalence of schistosomiasis had decreased dramatically by the time of the first impact assessment, with overall prevalence at just 3.5% (95% CI 2.6–4.7) (Table 3) and district level prevalence ranging from 0.0 to 45.0% (Figure 1; Supplementary Table 1). Every region had at least one district achieve zero prevalence of schistosomiasis. All regions, except for one, had prevalence of less than 10% with two regions having no schistosomiasis in the districts surveyed. (Figure 1; Supplementary Table 1).

#### Second schistosomiasis impact assessment (2021–2024)

A second impact evaluation was carried out in 1,146 schools sampled across 61 districts (334 sub-districts). In total, 29,924 SAC were sampled with 21,676 SAC providing a stool sample and 29,915 SAC providing a urine sample (Table 2). Overall, prevalence of any schistosomiasis was 6.8% (95% CI 6.1–7.6), which represents a 67.8% reduction from the baseline prevalence of 21.1% (Table 3). While only one district surveyed in the second impact assessment had zero prevalence of schistosomiasis, district level prevalence continued to decrease with a range of 0.0 to 21.5% (Figure 1; Supplementary Table 1).

At baseline and both impact assessments, the main species driving schistosomiasis prevalence is *S.haematobium* with a 67.0% reduction in prevalence from baseline (20.4%; 95% CI 16.4–25.2) to second impact assessment (6.7%; 95% CI 6.0–7.5) (Table 3). While prevalence of *S.mansoni* is relatively quite low, prevalence was still reduced by 88.1% from a prevalence of 1.0 (95% CI 0.6–1.9) at baseline to nearly zero at second impact assessment (0.12%; 95% CI 0.1–0.2) (Table 3).

#### Schistosomiasis intensity, gender, and age

Data on intensity of schistosomiasis infection, age, and gender was available only for the two impact assessments (2015 and 2021–2024) but not for baseline (2007–2010). Prevalence of any heavy intensity infection increased slightly from 1.6% (95% CI: 0.9–2.9) at first impact assessment to 2.1% (95% CI 1.7–2.6) at most recent impact assessment, representing a 31.1% increase, however, the difference is statistically insignificant (*p* = 0.39; Table 3). The increase was driven by a significant increase in *S.haematobium* heavy intensity infection from 1.4% (95% CI 0.8–2.4) at first impact assessment to 2.6% (95% CI 2.1–3.2; *p* = 0.04) (Table 3). Despite the increase in prevalence of heavy intensity infection between impact assessments, the proportion of heavy intensity schistosomiasis infections out of all infections decreased from 46.5% in first impact assessment to 32.3% in the most recent impact assessment.

**Figure 2:**
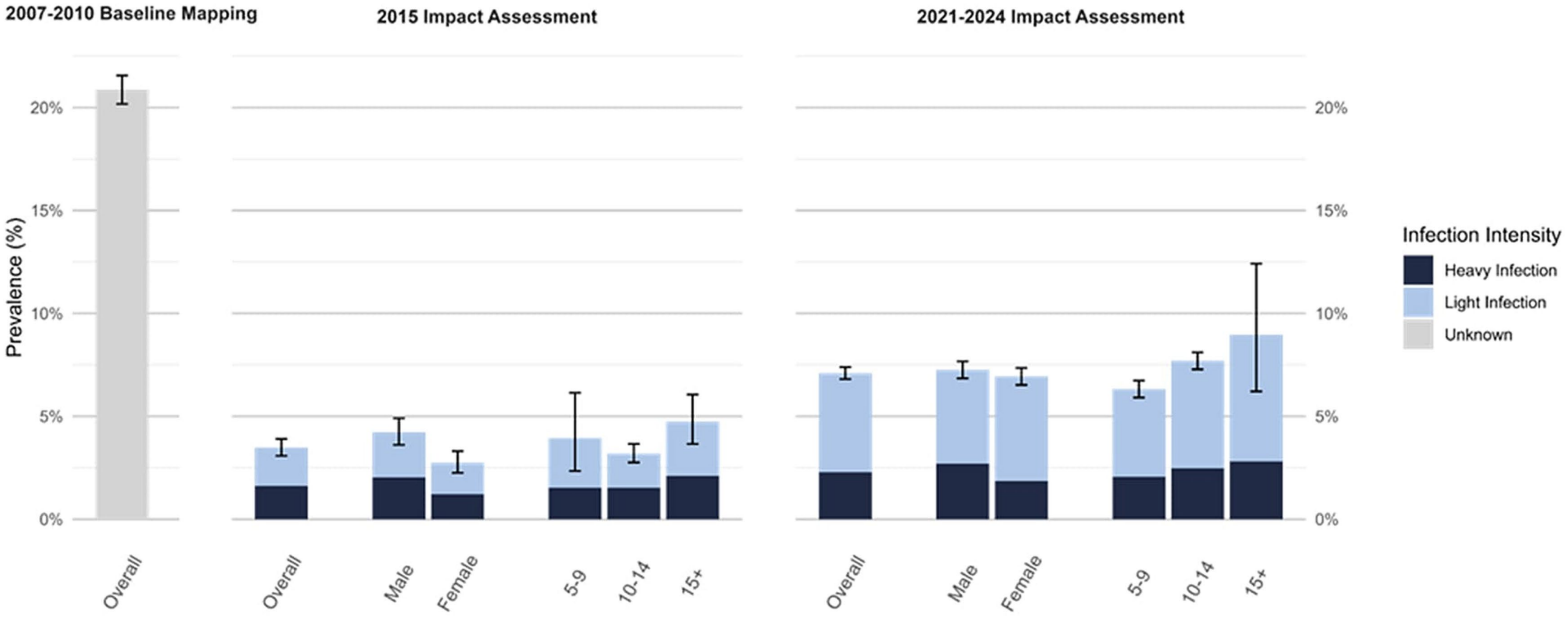
Schistosomiasis prevalence at first impact assessment (2015; *N* = 7,803) and most recent impact assessment (2021–2024; *N* = 29,924), overall and by intensity, gender, and age^*^. ^*^Intensity, gender, and age stratified prevalence values not available for 2007–2010 Baseline Mapping.

At the time of first impact assessment, males had a higher prevalence of schistosomiasis than females (4.2% vs. 2.7%, respectively), however, this difference narrowed by most recent impact assessment although prevalence in males was still slightly higher (7.3% vs. 6.9%, respectively). At both impact assessments, SAC reporting an age of 15 years or greater had the highest prevalence (4.7 and 9.0%, respectively) compared to SAC aged 10 to 14 years (3.2 and 7.7%, respectively) or aged 5 to 9 years (3.9 and 6.3%, respectively) (Figure 2). At first impact assessment, SAC aged 10 to 14 had the lowest prevalence (3.2%) but had the largest relative increase in prevalence by the time of second impact assessment (Figure 2).

## Discussion

Overall, there has been a significant reduction in overall prevalence of schistosomiasis in Ghana since the start of MDA in 2010 from 21.1% at baseline to 6.8% in 2024. This decrease in prevalence is largely due to the consistent therapeutic pressure in treating whole communities in high prevalence areas (>50%) and all SAC in areas of moderate and low endemicity. Despite this progress, infection appears to have plateaued in certain communities. These are often referred to as ‘persistent hotspots’ for schistosomiasis as they appear resilient to the MDA campaigns and maintain high prevalence and infection intensity (24). Reasons for persistence were not explored here but other studies in Ghana have shown that persistent schistosomiasis is not likely to be because of praziquantel resistance, but reinfection either due to high force of transmission or poor treatment compliance (25). Shifting from district-wide MDA to sub-district MDA will enable a more focused effort of interventions in such hotspots that will need additional efforts and future surveillance.

Although prevalence reductions are attributable to the impact of MDA, it is important to acknowledge the role of improved and more sensitive diagnostics and possible underestimation in earlier studies. Nevertheless, there are some limitations from comparing baseline, 2015 and recent (2021–2024) prevalence due to a change in the methods used. Firstly, the 2015 impact survey in Ghana surveyed a lot of the same schools as baseline, but the recent assessment sites were randomly selected, which could have masked a reduction in prevalence in the original schools. Secondly, the assessments at baseline and 2015 used one Kato-Katz slide but the more recent protocol used two slides (on a single stool sample), which would have increased the sensitivity of the diagnostic. The adoption of duplicate slides in addition to a quality control reading on 10% of all samples being re-read would have increased the accuracy of schistosomiasis diagnosis, particularly given the number of light infections. Third, the baseline and 2015 surveys were statistically powered to estimate district prevalence, but more recent surveys conducted between 2021 and 2024, were sampled to estimate subdistrict prevalence (17). Fourth, the most recent survey had an additional diagnostic using Haemastix®, as a proxy measure for *Schistosoma haematobium* and therefore are more useful for population studies and identifying high-risk communities rather than for individual patient diagnosis (26). This is a more sensitive diagnostic and may have increased more recent prevalence figures.

Despite these limitations, Ghana has moved from its initial goal of control of morbidity to elimination as a public health problem (EPHP). For schistosomiasis, the criterion for EPHP is defined by WHO as less than 1% prevalence of heavy-intensity infections (i.e., ≥50 *S. haematobium* eggs per 10 mL or ≥400 *S. mansoni* epg). As per the most recent surveys, EPHP has been achieved in 75.4% districts (78.3% subdistricts). There are limitations to EPHP as a benchmark of success, however, as it’s not only people with heavy-intensity infections, but nearly half of *S. mansoni* infections are missed using only a single stool sample of individuals with light-intensity. It seems, particularly as treatment is based on prevalence rather than intensity, there is a rationale to consider shifting the focus from schistosomiasis intensity EPHP targets to complementary indicators such as overall schistosomiasis prevalence (not just severe infections), average parasite load in the population, and reinfection rates to assess whether transmission persists even after MDA.

Currently, schistosomiasis control in Ghana is focused on preventive chemotherapy. The danger of MDA dependency, however, is that as control programs go on for long periods of time there is increasing drug fatigue and non-adherence to treatment in all age groups to varying extents. This is particularly apparent in men of ages 30+. Mathematical modeling has demonstrated the considerable impact of non-adherence on the success of MDA programmes to control NTDs. Long-term alternative interventions, such as investment to improve access to clean drinking water and sanitation, are important to sustain progress made by treatment in the longer term.

Finally, amidst this success seen in Ghana and other countries, particularly in West Africa, the WHO published new schistosomiasis recommendations in 2022 that encourage national control programs to shift from district to sub-district MDA to avoid mistreatment of areas within a district (6). The analysis of the Ghana data has shown a significant data gap at the sub-district level. The impact assessment conducted recently has been powered sufficiently to accurately determine the prevalence and appropriate treatment strategy for the sub-district, which resulted in a large sample size within each district. Over the next two years, Ghana will have closed this evidence gap to make a national shift in control strategy to the sub-district and hone the intervention efforts to the last mile endemic schistosomiasis areas. To maintain accurate monitoring and evaluation at the sub-district level, aggregating data at larger spatial scales may also mask heterogeneity in drug coverage at finer spatial scales and therefore indicators may remain above thresholds for EPHP in some areas. It is important therefore that a change in reporting systems is also adapted to record treatment coverage at sub-district (27).

## Conclusion

The Ghana Ministry of Health has made great strides in the control of schistosomiasis through the development and implementation of an efficient and comprehensive NTD control program supported by the GHS and the support of donors such as USAID. Since 2023, Ghana has shifted its intervention strategy to sub-district MDA in areas that have been re-assessed and therefore focus control efforts in those remaining areas of high transmission. The results of recent surveys have shown that MDA has been instrumental in reducing the burden of schistosomiasis in Ghana, but with the absence of sufficient water and sanitation access and the presence of large water bodies especially in the Volta region, elimination of these diseases as a public health problem or even to interrupt transmission will need additional interventions outside of MDA. Sustainable long-term interruption of NTD transmission will depend on future collaboration with the education and sanitation sector, environmental change, as well as strong surveillance plans.

## Data Availability

The datasets presented in this article are not readily available because the data generated and analyzed for this study is part of the Ghana Health Service Neglected Tropical Diseases Program. The dataset will be made available for reasonable requests only with permission from the Ministry of Health, Ghana. Requests to access the datasets should be directed to Joseph Opare, kwadwolarbiopare@gmail.com.
