# Supplementary material for: Schistosomiasis in Ghana from baseline to now: the impact of fifteen years of interventions
Source: Front Public Health. 2025 Jun 6;13:1554069. doi: 10.3389/fpubh.2025.1554069 (PMC12179181; doi:10.3389/fpubh.2025.1554069)
Supplement: Supplementary file 1 [file Table_1.DOCX]

**Supplementary Table 1: Regional schistosomiasis prevalence (*S.haematobium* and/or *S.mansoni*) and range of district level schistosomiasis prevalence by region at each survey**

|  | **2007-2010 Baseline** | | **2015 Impact Assessment** | | **2021-2024 Impact Assessment** | |
| --- | --- | --- | --- | --- | --- | --- |
| **Region** | **Any SCH Prevalence %** | **District Prevalence Range %**  **(# districts)** | **Any SCH Prevalence %** | **District Prevalence Range %**  **(# districts)** | **Any SCH Prevalence %** | **District Prevalence Range %**  **(# districts)** |
| Ahafo | 8.89 | 4.00 - 16.00 (5) | 0.00 | 0.00 - 0.00 (3) | - | - |
| Ashanti | 24.31 | 3.33 - 90.00 (21) | 1.91 | 0.00 - 16.00 (16) | 2.19 | 0.00 - 8.61 (18) |
| Bono | 7.15 | 0.00 - 30.00 (8) | 0.67 | 0.00 - 4.00 (5) | - | - |
| Bono East | 36.33 | 0.99 - 73.64 (8) | 10.80 | 0.00 - 45.00 (7) | - | - |
| Central | 26.57 | 0.00 - 58.00 (14) | 3.35 | 0.00 - 41.18 (12) | 4.95 | 1.94 - 10.28 (10) |
| Eastern | 36.47 | 3.33 - 98.33 (21) | 5.00 | 0.00 - 18.00 (14) | 6.88 | 1.39 - 21.67 (13) |
| Greater Accra | 30.25 | 4.00 - 66.00 (10) | 0.95 | 0.00 - 5.56 (7) | - | - |
| Northeast | 2.87 | 0.00 - 6.67 (4) | 0.00 | 0.00 - 0.00 (3) | - | - |
| Northern | 5.82 | 0.00 - 25.63 (13) | 2.67 | 0.00 - 8.00 (8) | 11.82 | 11.86 (1) |
| Oti | 41.11 | 8.00 - 86.00 (5) | 4.86 | 0.00 - 11.00 (4) | 18.49 | 13.78 - 17.74 (2) |
| Savannah | 5.86 | 0.00 - 29.09 (6) | 0.57 | 0.00 - 2.00 (4) | 4.31 | 2.50 - 6.11 (2) |
| Upper East | 25.13 | 6.00 - 70.00 (9) | 4.99 | 0.00 - 26.09 (8) | 6.20 | 1.94 - 12.78 (3) |
| Upper West | 8.28 | 0.59 - 35.45 (7) | 1.50 | 0.00 - 7.00 (6) | - | - |
| Volta | 24.48 | 6.67 - 53.64 (7) | 2.22 | 0.00 - 9.00 (7) | 4.44 | 0.83 - 18.18 (9) |
| Western | 21.42 | 6.00 - 44.12 (9) | 4.62 | 0.00 - 25.00 (5) | 6.94 | 6.94 (1) |
| Western North | 16.34 | 2.00 - 37.76 (7) | 6.57 | 0.00 - 12.00 (5) | 4.44 | 2.50 - 6.39 (2) |
